# Comparison of Proximal Tibiofibular Joint Detachment with Tibial-Sided Osteotomy for Fibular Untethering in Lateral Closing-Wedge High Tibial Osteotomy: A Cadaveric Study

**DOI:** 10.3390/medicina61010161

**Published:** 2025-01-19

**Authors:** Ryu Kyoung Cho, Keun Young Choi, Dai-Soon Kwak, Man Soo Kim, Yong In

**Affiliations:** 1 Department of Orthopaedic Surgery, Seoul St. Mary’s Hospital, College of Medicine, The Catholic University of Korea, Seoul 06591, Republic of Korea; dreamer1222@naver.com (R.K.C.); heaxagon@hanmail.net (K.Y.C.); kms3779@naver.com (M.S.K.); 2 Catholic Institute for Applied Anatomy, Department of Anatomy, College of Medicine, The Catholic University of Korea, Seoul 06591, Republic of Korea; daisoon@catolic.ac.kr

**Keywords:** lateral closing-wedge high tibial osteotomy, proximal tibiofibular joint, fibular shaft osteotomy, proximal tibiofibular joint detachment, tibial-sided osteotomy

## Abstract

*Background and Objectives:* Proximal tibiofibular joint detachment (PTFJD) is a fibular untethering procedure during lateral closing-wedge high tibial osteotomy (LCWHTO) for varus knee osteoarthritis. However, the PTFJD procedure is technically demanding, and confirmation of clear joint separation is not straightforward. The aim of this study was to compare the degree of completion and safety of PTFJD versus tibial-sided osteotomy (TSO); this latter procedure is our novel technique for fibular untethering during LCWHTO. *Materials and Methods:* Sixteen fresh frozen cadaver knees from eight cadavers were included in the study. Among the eight pairs of knees, one knee was randomly assigned to undergo PTFJD and the other knee to undergo TSO, which separates the fibula by osteotomizing the lateral cortex of the proximal tibia at the medial side of the proximal tibiofibular joint for fibular untethering during LCWHTO. After each procedure with LCWHTO, the posterior compartment of each knee was dissected to compare the degree of procedural completion and the distance from the posterior detachment or osteotomy site to posterior neurovascular structures between PTFJD and TSO groups. The pass-through test crossing the separation site from anterior to posterior using an osteotome was also performed to evaluate the protective effect of the muscular structures of the posterior compartment. *Results:* In the PTFJD group, four of eight cases (50%) showed fibular head fractures rather than division of the proximal tibiofibular joint. In contrast, in all TSO cases, the lateral cortex of the proximal tibia was clearly osteotomized from the medial side of the posterior proximal tibiofibular joint. Distances from the posterior detachment or osteotomy site to the common peroneal nerve, popliteal artery, and anterior tibial artery in the PTFJD and TSO groups were 20.8 ± 3.3 mm and 22.9 ± 3.6 mm (*p* = 0.382), 11.0 ± 2.4 mm and 9.8 ± 2.8 mm (*p* = 0.382), and 14.8 ± 1.9 mm and 14.9 ± 2.5 mm (*p* = 0.721), respectively. In the pass-through test, an osteotome was able to pass anteriorly to posteriorly in all eight PTFJD group cases. However, the osteotome was blocked posteriorly by the popliteus muscle in the TSO group cases, indicating protection of posterior neurovascular structures during the TSO procedure. *Conclusions:* TSO, a novel fibular untethering procedure for LCWHTO, resulted in clear separation of the fibula from the lateral tibial cortex, and protection of posterior neurovascular structures by the popliteus muscle during the procedure. We anticipate that our novel surgical technique will provide more clear-cut and safer fibular untethering for LCWHTO.

## 1. Introduction

High tibial osteotomy (HTO) is a joint-preserving procedure that is widely accepted as an effective surgical option for young patients with isolated medial compartment osteoarthritis (OA) in the varus knee [1]. Although medial opening-wedge high tibial osteotomy (MOWHTO) using a modern locking plate system has become popular [2,3], lateral closing-wedge high tibial osteotomy (LCWHTO) has obvious benefits when a large correction is needed [4]. It has the strengths of quicker rehabilitation, better initial stability, no need for a bone graft, and maintenance of patellar position and tibial slope [5,6,7,8]. Moreover, better clinical outcomes have also been reported in patients following LCWHTO compared to MOWHTO [9].

However, LCWHTO has the disadvantage that it requires a fibular untethering procedure for osteotomy gap closing such as fibular shaft osteotomy (FSO) or proximal tibiofibular joint detachment (PTFJD) [10,11,12,13]. In FSO, an additional incision is required to perform osteotomy of the fibular shaft, which can lead to non-union at the osteotomy site [14], and there is the risk of fatality due to peroneal nerve injury [15,16]. In contrast, peroneal nerve injury is uncommon during PTFJD [11,17]. However, confirmation of clear proximal tibiofibular joint (PTFJ) separation is not straightforward, especially because the posterior part is a blind spot. In normal PTFJD procedures, repeated osteotomy is performed until PTFJ separation is confirmed.

The anatomy of the PTFJ is complex. The anterior capsule of the PTFJ is stabilized by the anterior tibiofibular ligament, which consists of three broad bands, while the posterior joint capsule is supported by the posterior proximal tibiofibular ligament, which consists of two thick bands [18,19]. This complicated anatomy can cause incomplete separation of the PTFJ during LCWHTO. In addition, although the incidence is low, popliteal neurovascular bundle injuries have been reported after LCWHTO [20,21,22].

Given this uncertainty and these complications of PTFJD, our group devised an alternative fibular untethering procedure, tibial-sided osteotomy (TSO), which separates the fibula and tibial bone fragment from the lateral cortex of the proximal tibia nearby the medial side of PTFJ. The purpose of this study was to compare the degree of completion and safety of PTFJD and TSO during LCWHTO.

## 2. Materials and Methods

In our study, 16 knees from eight fresh cadavers (three males and five females) were used. Mean age was 80.4 ± 9.1 years (range, 62–93 years). All knees were grossly intact with no history of injury or surgery to the knee joint or PTFJ. Specimens were maintained in a frozen state at −20 °C and allowed to thaw at room temperature for 24 h before dissection. One knee of each cadaver was randomly assigned to the PTFJD group and the other knee to the TSO group by simple randomization. After the designated fibular untethering procedure, LCWHTO was performed in all knees. All surgeries were carried out by two orthopaedic surgeons (Drs. RKC and KYC) together to reduce technical error. This study was approved by the Institutional Cadaver Research Committee of our Institution (research number: MC23EADI0064, IRB review date: 21 July 2023). All donors or their authorized representatives provided written informed consent for use of the cadaver and agreed to the utilization of related materials in the research.

**Figure 1:**
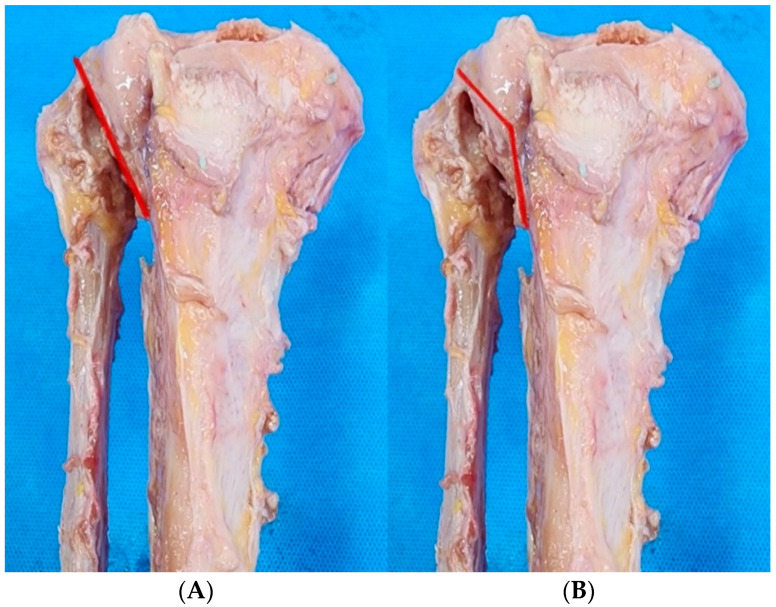
(**A**) Proximal tibiofibular joint detachment and (**B**) tibial-sided osteotomy. The red lines indicate the sites of fibular untethering following each of the two procedures.

All surgical techniques were performed on the cadaver identically to how they would have been performed in a living patient. An oblique longitudinal incision was made 1 cm below the fibular head on the anterior aspect of the knee toward the tibial tuberosity, and an additional incision was made in the lower-leg fascia. Using a knife and a periosteal elevator, the periosteum was dissected from below Gerdy’s tubercle to below the outer border of the tibial tuberosity. The tibialis anterior muscle and periosteum were retracted laterally to prevent neurovascular injury. Both PTFJD and TSO were performed at a knee flexion angle of 90° to reduce the risk of peroneal nerve injury. PTFJD was carried out using the traditional method. After retracing the tibialis anterior muscle, the anterior proximal tibiofibular ligament (PTFL) was identified. The anterior PTFL was divided and the PTFJ was detached using an osteotome repeatedly toward the posterior PTFL until the tibia and fibula were separated under the image intensifier (Figure 1A). For TSO, a Kirschner wire was inserted at a position 5 mm medially from the lateral cortex of the proximal tibia near the PTFJ. After creating a chevron-shaped saw pattern in the anterior tibial cortex along the inserted Kirschner wire, osteotomy to the posterior tibial cortex was commenced using an osteotome (Figure 1B). The TSO procedure was completed after confirming that the thin lateral tibial bone fragment that attached to the PTFJ was separated from the tibia under the image intensifier.

Regardless of the PTFJ separation method, LCWHTO was performed in the same manner with the knee in extension. All LCWHTOs were fixed using a TOMOFIX^TM^ Lateral High Tibia Plate (DePuy Synthes, Zuchwil, Switzerland). The correction angle goal was set to 10° regardless of the varus deformity of the cadaver. After checking the position of the plate where four screws each could be fixed proximally and distally, two anterior and posterior Kirschner wires were inserted toward the medial target point (20 mm distally to the proximal medial tibial joint surface) parallel to the tibial plateau. Subsequently, two additional anterior and posterior Kirschner wires were inserted 10 mm below the previously inserted Kirschner wires. A biplanar osteotomy, which consisted of a tibial tubercle osteotomy and upper and lower tibial osteotomies, was performed using a saw and chisels. After removing the bone fragment, valgus force was applied to close the gap between the upper and lower tibial cut surfaces. While applying valgus force to maintain the contact, eight screws were fixed.

To compare the degree of completion of each procedure, anatomic dissection of the posterior compartment of the knee was performed. Skin and subcutaneous tissue were dissected through an inverted L-shaped incision, exposing the underlying popliteal fossa. Lateral posterior structures were dissected to find the PTFJ and common peroneal nerve in its location beneath the biceps femoris. After laterally retracting the common peroneal nerve, the origin of the lateral head of the gastrocnemius was identified and sacrificed to provide broad exposure of the deep structures on the lateral aspect of the posterior joint, including the arcuate ligament and popliteus muscle. After retracting the popliteus muscle, the degree of procedure completion could be assessed. The objective was clear detachment of the posterior PTFJ in the PTFJD group or clear separation of the bone fragment from the posterolateral cortex of the tibia in the TSO group.

The safety of each procedure was evaluated by measuring the positional relationship between the detachment or osteotomy site and posterior neurovascular structures of the knee in millimeters using a surgical ruler. In the PTFJD and TSO groups, the actual posterior detachment or osteotomy site served as the reference point for measuring distances to posterior neurovascular structures. Distances from each reference point to the common peroneal nerve, popliteal artery, and anterior tibia artery were measured.

The pass-through test was performed to identify the protective effect of the popliteus muscle in each procedure. An osteotome was passed through the detachment or osteotomy site anteriorly to posteriorly to evaluate whether the osteotome was stopped by the popliteus muscle or not.

### Statistical Analyses

All calculated data are shown as means with standard deviations using the SPSS statistical software (SPSS 28; SPSS Inc., Chicago, IL, USA). Chi-square and Fisher’s exact test were used to determine the significance of differences in categorical or dichotomized data between groups, while t-tests and Mann–Whitney U tests were used to determine the significance of differences in continuous variables between groups. Statistical significance was accepted at *p* < 0.05 for all analyses.

## 3. Results

**Figure 2:**
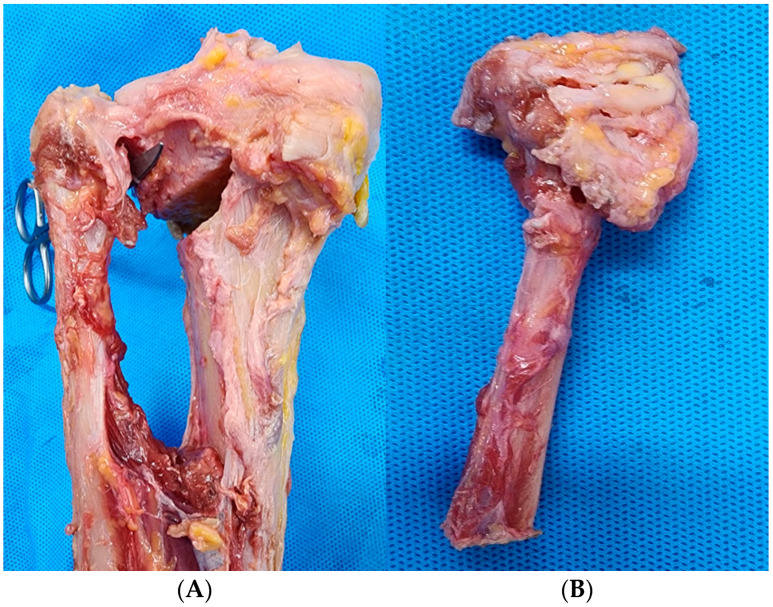
(**A**) Posterior aspect of a knee after PTFJD showing clear separation of the PTFJ. (**B**) A fibular head fracture case involving articular cartilage after PTFJD.

**Figure 3:**
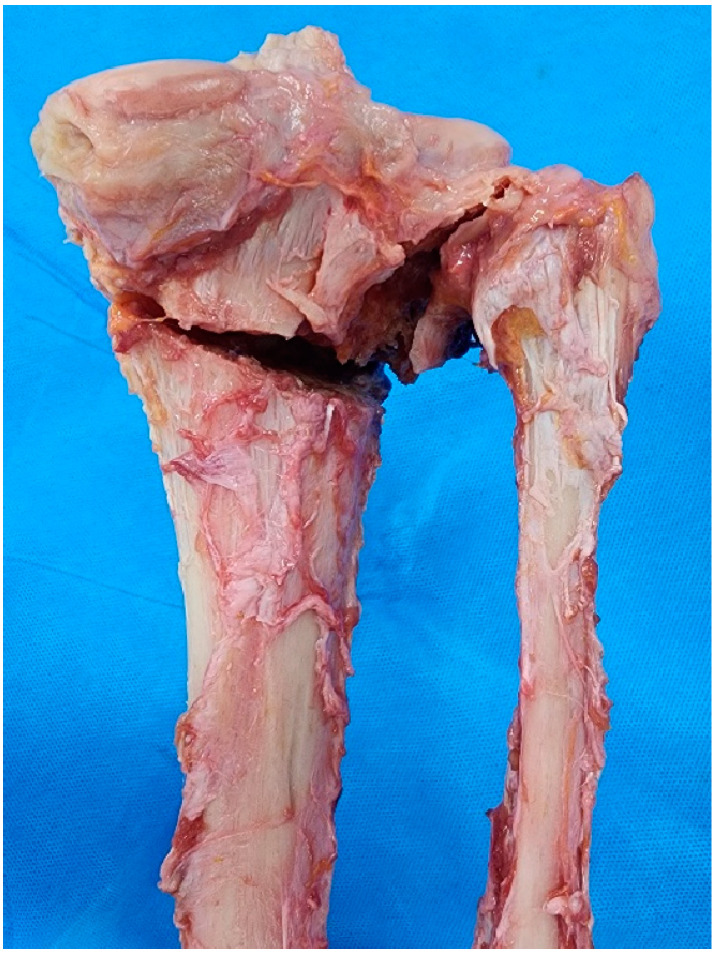
Posterior aspect of a knee showing separation of fibula with tibial bone fragment after TSO.

PTFJD and TSO with LCWHTO were completed without incomplete closing in all knees. The degree of completion of each procedure was investigated by anatomic dissection of the posterior compartment of the knee. In the PTFJD group, the posterior PTFJ was clearly separated in 50% of cases (Figure 2A). However, in the remaining four cases, a fibular head fracture was observed after PTFJD rather than the intended PTFJ separation (Figure 2B). In contrast, posterior osteotomy was consistently observed at the posterolateral cortex of the tibia in all eight cases (100%) (Figure 3). In other words, the degree of procedural completion was more reliable in the TSO group (100%) than in the PTFJD group (50%) (*p* = 0.21). Including four fibular head fracture cases, a total of five cases showed articular surface injury of the fibular head in the PTFJD group (62.5%). However, the PTFJ was intact in all TSO cases (100%) (*p* = 0.007).

Table 1 shows the average distances from the posterior detachment or osteotomy site to the common peroneal nerve, popliteal artery, and anterior tibial artery. Distances in the PTFJD group from the posterior detachment site to the common peroneal nerve and anterior tibial artery tended to be smaller, but without statistical significance (*p* = 0.382 and *p* = 0.721, respectively). Conversely, the popliteus artery was closer to the osteotomy site in the TSO group, again without statistical significance (*p* = 0.382).

**Figure 4:**
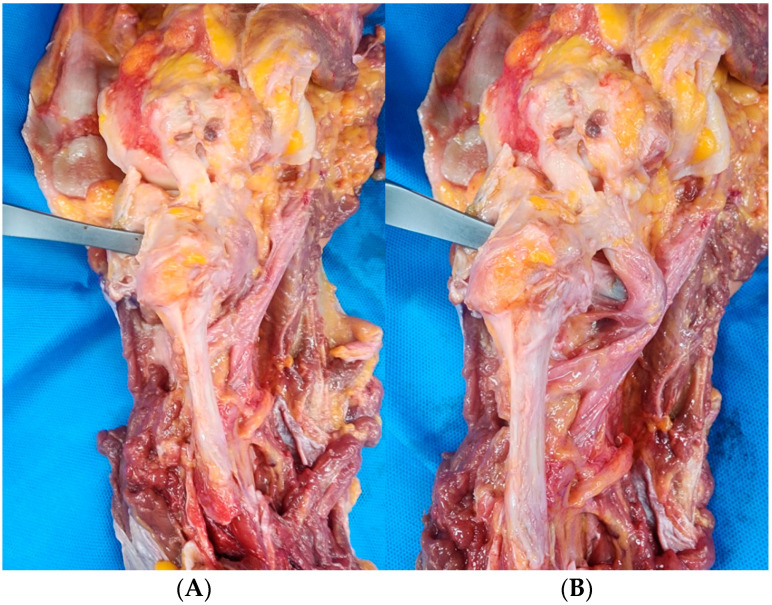
The protective effect of the popliteus muscle. (**A**) Before entry of the osteotome; (**B**) protected appearance after penetration of the osteotome.

The popliteus muscle covers the posterior aspect of the tibia but does not extend to cover the posterior PTFJ. Substantial differences between the two surgical techniques were uncovered. In the case of PTFJD, because of the absence of popliteus muscle coverage of the posterior PTFJ, an osteotome passed through the PTFJ without popliteus muscle protection in all eight cases. In contrast, the osteotome was blocked by the popliteus muscle while passing the osteotomy site in all eight cases, indicating that the popliteus muscle plays a crucial role protecting posterior neurovascular structures during TSO, a fibular untethering procedure (Figure 4).

## 4. Discussion

As mentioned in the introduction, HTO has been considered an effective surgical option for patients with medial knee osteoarthritis (OA) with varus deformity [1]. Moreover, numerous studies report high levels of patient satisfaction and excellent clinical outcomes following HTO [23], with significant advancements continuously being made in the technique. Recently, HTO has increasingly been performed in combination with various cartilage procedures, such as microfracture [24], bone marrow aspirate concentrate (BMAC) [25], human umbilical cord blood-derived mesenchymal stem cells (hUCB-MSC) [26], and collagen augmentation [27]. When HTO is combined with cartilage procedures, second-look arthroscopy often reveals cartilage regeneration, accompanied by excellent clinical outcomes [28]. Takeuchi et al. [29] introduced a new hybrid closing HTO to complement the shortcomings of traditional LCWHTO, and fibular untethering is also required in this technique. The TSO procedure introduced in this study is expected to perform fibular untethering more precisely and safely.

The most important finding of this study is that our novel surgical technique, namely TSO, allowed for the procedural completion of fibular untethering by preserving the PTFJ. In our conventional PTFJD procedure, there was articular surface damage of the fibular head in 62.5% of cases. In addition, the TSO procedure was safer than PTFJD because of the protective effect of the popliteus muscle, as the separation point is more medial than in the PTFJD procedure.

Peroneal nerve dysfunction is a serious complication that can occur after LCWHTO [30,31], with reported rates of symptomatic peroneal nerve injury ranging between 3.3 and 20% [16,30]. While unlikely, there exists the possibility of peroneal nerve damage if the osteotomy is conducted with bias toward the fibula. The average distance from the division site of the common peroneal nerve into the superficial peroneal nerve and deep peroneal nerve to the posterolateral tip of the fibular head has been reported to be 20.7 mm [32]. Although the reference points were different in the study discussed above compared to our study, we observed that the distance from the posterior osteotomized site to the common peroneal nerve was similar in both the PTFJD and TSO groups. The distances mentioned above suggest a potential risk if PTFJD is performed biased toward the fibula. However, TSO has the advantage of reducing the possibility of peroneal nerve damage compared to PTFJD, as the osteotomy is directed toward the tibia. Although we did not find a statistically significant difference between the two groups due to our limited sample size given that this was a cadaveric study, the distance from the common peroneal nerve was numerically greater in the TSO group than in the PTFJD group. Therefore, additional research with a larger sample size is needed to determine if this difference is meaningful.

As mentioned above, LCWHTO is a useful surgical treatment to correct isolated medial OA with varus knee requiring large correction [4]. However, this procedure can result in injury of the popliteal neurovascular bundle, with reported rates of injury around the popliteal artery of between 0.4% and 9.8% [30,33]. Ricardo et al. reported a case of popliteal artery injury after LCWHTO leading to secondary neurological injury [20]. Although popliteal neurovascular injury is rare, this complication can be catastrophic. In our study, the TSO group was closer to the popliteal artery than the PTFJD group; this is likely because the posterior osteotomy site of the tibia was positioned more medially. As shown in the pass-through test results of this study, the popliteus muscle had a protective effect in the TSO procedure. Therefore, we believe that there are no safety concerns when performing TSO if the posterior osteotomy site of the tibia is located medially.

The anterior tibial artery, one of the terminal branches of the popliteal artery, is vulnerable during LCWHTO. Injury of the anterior tibial artery may occur when performing PTFJD using an osteotome. In fact, one study [22] reported a pseudoaneurysm due to anterior tibial arterial injury after LCWHTO. As mentioned in the previous paragraph, PTFJD can cause damage to the fibular head rather than achieving precise division of the PTFJ. Furthermore, even if a surgeon precisely separates the PTFJ, the risk of anterior tibial arterial injury may be increased. In our study, we found no significant difference in distance to the anterior tibial artery between the TSO and PTFJD groups. However, using our new technique, the probability of anterior tibial arterial injury was significantly reduced due to the protective effect of the popliteus muscle. To the best of our knowledge, although many surgeons have performed FSO or PTFJD in LCWHTO, no previous study has systematically evaluated the role of the popliteus muscle. Elucidation of the protective effect of the popliteus muscle would hopefully allow for safer PTFJ manipulation when performing LCWHTO.

PTFJ has been called the fourth compartment of the knee joint [17,20,34]. Although it is a small joint, if there is a pathologic lesion, it can cause lateral knee pain. In fact, Murat et al. reported that PTFJ pathologies result in lateral knee pain [35,36]. The case report of Enrique et al. stated that injury of the PTFJ caused unexplained lateral knee pain after LCWHTO [37]. On bone SPECT CT to detect arthritic changes, “hot” uptake by the PTFJ was noted along with iatrogenic perforation of the screw; osteoarthritis subsequently developed. This suggests that injury of the PTFJ, including PTFJ articular cartilage, can progress to osteoarthritis. There were no cases of PTFJ injury, including the cartilage, after TSO. Conversely, PTFJ cartilage injury was identified in five of eight knees (62.5%) after PTFJD (*p* = 0.007). In other words, by performing osteotomy biased toward the tibia without causing injury to the PTFJ, TSO is likely to be associated with a lower likelihood of experiencing lateral knee pain due to PTFJ injury after LCWHTO than PTFJD.

The two representative methods of proximal tibial osteotomy are MOWHTO and LCWHTO. Many studies have reported that there is no significant difference between the two techniques in terms of clinical outcomes or radiographic alignment [38]. As mentioned in the introduction, with the development of the locking plate system, many surgeons are preferring MOWHTO because it is relatively easier to perform than LCWHTO [2,3], and it has several advantages [5]. However, although MOWHTO has many merits and LCWHTO is technically challenging, it is crucial to carefully select the appropriate osteotomy based on the specific condition of the patient. For example, if the correction angle is large, MOWHTO may result in a leg length longer than that of the contralateral limb [4], making LCWHTO a more suitable option. In the case of MOWHTO, patella baja may occur [39], and rotational changes can lead to anterior knee pain. Therefore, LCWHTO may be more advantageous for patients with patellofemoral arthritis [40]. Additionally, the choice between the two techniques should be guided by the presence or absence of ACL or PCL deficiency [41], ensuring appropriate selection for each patient. To achieve optimal clinical outcomes, the selection between the two surgical methods should be tailored to the patient’s specific condition. Therefore, our new surgical technique can be appropriately utilized when LCWHTO is required in the situations mentioned above.

This study had several limitations, mainly due to our utilization of a cadaveric model. First, the preparation of each specimen may not have precisely replicated natural knee conditions. Second, only cadavers of Korean origin were utilized. Hence, there is the potential for ethnic selection bias in the data. Third, the majority of specimens did not represent knees with isolated medial compartment osteoarthritis requiring more than 10 degrees of correction. Fourth, the number of cadaver samples was relatively small. Fifth, as the correction angle of 10 degrees was uniformly applied to all cadavers, the correction was not individualized to the unique characteristics of each cadaver’s knee joint; thus, diversity was not adequately taken into account.

## 5. Conclusions

We demonstrated that our innovative surgical technique, TSO, which involves tibia-biased osteotomy during PTFJ manipulation in LCWHTO, is a more precise and safer procedure than conventional PTFJD. We attribute this not only to the tibia-biased osteotomy, which enhances the efficacy of procedural completion and diminishes the likelihood of common peroneal nerve damage, but also to the protective effect of the popliteus muscle, which safeguards the posterior neurovascular structures. TSO offers a more precise and safer approach to manipulating the PTFJ during fibular untethering in LCWHTO than PTFJD.

## Figures and Tables

**Table 1:** Distances to posterolateral neurovascular structures from the posterior detachment or osteotomy site after PTFJD and TSO.

	PTFJ Detachment (n = 8)	Tibia-Sided Osteotomy (n = 8)	*p*-Value
Distance to common peroneal nerve (mm)	20.8 ± 3.3	22.9 ± 3.6	0.382
Distance to popliteus artery (mm)	11.0 ± 2.4	9.8 ± 2.8	0.382
Distance to anterior tibial artery (mm)	14.8 ± 1.9	14.9 ± 2.5	0.721

PTFJD: proximal tibiofibular joint detachment. PTFJ: proximal tibiofibular joint. PTFL: proximal tibiofibular ligament. TSO: tibial-side osteotomy.

## Data Availability

The data presented in this study are available on request from the corresponding author.

## References

[1] Gomoll A.H. (2011). High tibial osteotomy for the treatment of unicompartmental knee osteoarthritis: A review of the literature, indications, and technique. Phys. Sportsmed..

[2] Hui C., Salmon L.J., Kok A., Williams H.A., Hockers N., van der Tempel W.M., Chana R., Pinczewski L.A. (2011). Long-term survival of high tibial osteotomy for medial compartment osteoarthritis of the knee. Am. J. Sports Med..

[3] Miller B.S., Joseph T.A., Barry E.M., Rich V.J., Sterett W.I. (2007). Patient satisfaction after medial opening high tibial osteotomy and microfracture. J. Knee Surg..

[4] Kim J.I., Kim B.H., Lee K.W., Lee O., Han H.S., Lee S., Lee M.C. (2016). Lower Limb Length Discrepancy After High Tibial Osteotomy: Prospective Randomized Controlled Trial of Lateral Closing Versus Medial Opening Wedge Osteotomy. Am. J. Sports Med..

[5] Murray R., Winkler P.W., Shaikh H.S., Musahl V. (2021). High Tibial Osteotomy for Varus Deformity of the Knee. J. Am. Acad. Orthop. Surg. Glob. Res. Rev..

[6] Amendola A., Bonasia D.E. (2010). Results of high tibial osteotomy: Review of the literature. Int. Orthop..

[7] Brouwer R.W., Bierma-Zeinstra S.M., van Raaij T.M., Verhaar J.A. (2006). Osteotomy for medial compartment arthritis of the knee using a closing wedge or an opening wedge controlled by a Puddu plate. A one-year randomised, controlled study. J. Bone Jt. Surg. Br..

[8] Gooi S.G., Chan C.X.Y., Tan M.K.L., Lim A.K.S., Satkunanantham K., Hui J.H.P. (2017). Patella Height Changes Post High Tibial Osteotomy. Indian J. Orthop..

[9] van Egmond N., van Grinsven S., van Loon C.J., Gaasbeek R.D., van Kampen A. (2016). Better clinical results after closed- compared to open-wedge high tibial osteotomy in patients with medial knee osteoarthritis and varus leg alignment. Knee Surg. Sports Traumatol. Arthrosc..

[10] Kurosaka M., Tsumura N., Yoshiya S., Matsui N., Mizuno K. (2000). A new fibular osteotomy in association with high tibial osteotomy (a comparative study with conventional mid-third fibular osteotomy). Int. Orthop..

[11] Aydoğdu S., Yercan H., Saylam C., Sur H. (1996). Peroneal nerve dysfunction after high tibial osteotomy. An anatomical cadaver study. Acta Orthop. Belg..

[12] Coventry M.B. (1973). Osteotomy about the knee for degenerative and rheumatoid arthritis. J. Bone Jt. Surg. Am..

[13] Soejima O., Ogata K., Ishinishi T., Fukahori Y., Miyauchi R. (1994). Anatomic considerations of the peroneal nerve for division of the fibula during high tibial osteotomy. Orthop. Rev..

[14] Ramanoudjame M., Vandenbussche E., Baring T., Solignac N., Augereau B., Gregory T. (2012). Fibular nonunion after closed-wedge high tibial osteotomy. Orthop. Traumatol. Surg. Res..

[15] Preston C.F., Fulkerson E.W., Meislin R., Di Cesare P.E. (2005). Osteotomy about the knee: Applications, techniques, and results. J. Knee Surg..

[16] Wootton J.R., Ashworth M.J., MacLaren C.A. (1995). Neurological complications of high tibial osteotomy--the fibular osteotomy as a causative factor: A clinical and anatomical study. Ann. R. Coll. Surg. Engl..

[17] Özcan Ö., Eroglu M., Boya H., Kaya Y. (2017). Proximal tibiofibular joint pain versus peroneal nerve dysfunction: Clinical results of closed-wedge high tibial osteotomy performed with proximal tibiofibular joint disruption. Knee Surg. Sports Traumatol. Arthrosc..

[18] Marchetti D.C., Moatshe G., Phelps B.M., Dahl K.D., Ferrari M.B., Chahla J., Turnbull T.L., LaPrade R.F. (2017). The Proximal Tibiofibular Joint: A Biomechanical Analysis of the Anterior and Posterior Ligamentous Complexes. Am. J. Sports Med..

[19] Scarciolla L., Herteleer M., Turquet E., Badr S., Demondion X., Jacques T., Cotten A. (2021). Anatomical study of the proximal tibiofibular ligaments using ultrasound. Insights Imaging.

[20] Santos-Pereira R., Aleixo C., Bernardes M., Costa A., Fontes-Lebre J., Carvalho P.J. (2019). Popliteal Artery Pseudoaneurysm after Closing Wedge High Tibial Osteotomy. J. Orthop. Case Rep..

[21] Vena G., D’Adamio S., Amendola A. (2013). Complications of osteotomies about the knee. Sports Med. Arthrosc. Rev..

[22] Sawant M.R., Ireland J. (2001). Pseudo-aneurysm of the anterior tibial artery complicating high tibial osteotomy—A case report. Knee.

[23] Cho R.K., Kim M.S., Choi K.Y., In Y. (2024). Is severe medial knee osteoarthritis a risk factor for dissatisfaction following medial open-wedge high tibial osteotomy in patients 55 years of age or younger?. Orthop. Traumatol. Surg. Res..

[24] Lee O.S., Lee S.H., Mok S.J., Lee Y.S. (2019). Comparison of the regeneration of cartilage and the clinical outcomes after the open wedge high tibial osteotomy with or without microfracture: A retrospective case control study. BMC Musculoskelet. Disord..

[25] Keeling L.E., Belk J.W., Kraeutler M.J., Kallner A.C., Lindsay A., McCarty E.C., Postma W.F. (2022). Bone Marrow Aspirate Concentrate for the Treatment of Knee Osteoarthritis: A Systematic Review. Am. J. Sports Med..

[26] Park Y.B., Ha C.W., Lee C.H., Yoon Y.C., Park Y.G. (2017). Cartilage Regeneration in Osteoarthritic Patients by a Composite of Allogeneic Umbilical Cord Blood-Derived Mesenchymal Stem Cells and Hyaluronate Hydrogel: Results from a Clinical Trial for Safety and Proof-of-Concept with 7 Years of Extended Follow-Up. Stem Cells Transl. Med..

[27] Kim M.S., Koh I.J., Choi Y.J., Pak K.H., In Y. (2017). Collagen Augmentation Improves the Quality of Cartilage Repair After Microfracture in Patients Undergoing High Tibial Osteotomy: A Randomized Controlled Trial. Am. J. Sports Med..

[28] Han J.H., Jung M., Chung K., Jung S.H., Choi C.H., Kim S.H. (2024). Effects of concurrent cartilage procedures on cartilage regeneration in high tibial osteotomy: A systematic review. Knee Surg. Relat. Res..

[29] Takeuchi R., Ishikawa H., Miyasaka Y., Sasaki Y., Kuniya T., Tsukahara S. (2014). A novel closed-wedge high tibial osteotomy procedure to treat osteoarthritis of the knee: Hybrid technique and rehabilitation measures. Arthrosc. Tech..

[30] Atrey A., Morison Z., Tosounidis T., Tunggal J., Waddell J.P. (2012). Complications of closing wedge high tibial osteotomies for unicompartmental osteoarthritis of the knee. Bone Jt. Res..

[31] Aydogdu S., Cullu E., Araç N., Varolgüneş N., Sur H. (2000). Prolonged peroneal nerve dysfunction after high tibial osteotomy: Pre- and postoperative electrophysiological study. Knee Surg. Sports Traumatol. Arthrosc..

[32] Grechenig P., Hohenberger G., Maier M., Grechenig C., Holweg P., Vielgut I., Weiglein A., Gänsslen A. (2021). The articular branch of the peroneal nerve to the proximal tibiofibular joint descends at a mean height of approximately 18 mm distal to the postero-lateral tip of the fibular head. Knee Surg. Sports Traumatol. Arthrosc..

[33] Efe T., Ahmed G., Heyse T.J., Boudriot U., Timmesfeld N., Fuchs-Winkelmann S., Ishaque B., Lakemeier S., Schofer M.D. (2011). Closing-wedge high tibial osteotomy: Survival and risk factor analysis at long-term follow up. BMC Musculoskelet. Disord..

[34] Gil-Melgosa L., Valentí A., Suárez Á., Montiel V. (2020). Proximal tibiofibular joint changes after closed-wedge high tibial osteotomy. Are they relevant?. Knee.

[35] Bozkurt M., Yilmaz E., Akseki D., Havitcioğlu H., Günal I. (2004). The evaluation of the proximal tibiofibular joint for patients with lateral knee pain. Knee.

[36] Semonian R.H., Denlinger P.M., Duggan R.J. (1995). Proximal tibiofibular subluxation relationship to lateral knee pain: A review of proximal tibiofibular joint pathologies. J. Orthop. Sports Phys. Ther..

[37] Testa E.A., Haeni D.L., Behrens G., Hirschmann M.T. (2014). Unexplained proximal tibiofibular joint pain after high tibial osteotomy. Indian J. Orthop..

[38] Duivenvoorden T., Brouwer R.W., Baan A., Bos P.K., Reijman M., Bierma-Zeinstra S.M., Verhaar J.A. (2014). Comparison of closing-wedge and opening-wedge high tibial osteotomy for medial compartment osteoarthritis of the knee: A randomized controlled trial with a six-year follow-up. J. Bone Jt. Surg. Am..

[39] Bin S.I., Kim H.J., Ahn H.S., Rim D.S., Lee D.H. (2016). Changes in Patellar Height After Opening Wedge and Closing Wedge High Tibial Osteotomy: A Meta-analysis. Arthroscopy.

[40] Kim J.I., Kim B.H., Han H.S., Lee M.C. (2020). Rotational Changes in the Tibia After High Tibial Valgus Osteotomy: A Comparative Study of Lateral Closing Versus Medial Opening Wedge Osteotomy. Am. J. Sports Med..

[41] Agostinone P., Lucidi G.A., Dal Fabbro G., Di Paolo S., Pagano A., Viotto M., Neri M.P., Grassi A., Zaffagnini S. (2023). Satisfactory long-term outcomes for combined anterior cruciate ligament reconstruction and lateral closing wedge high tibial osteotomy. Knee Surg. Sports Traumatol. Arthrosc..

